# Cognitive impairment in migraine: A systematic review

**DOI:** 10.1590/S1980-57642012DN06020002

**Published:** 2012

**Authors:** Caroline Martins de Araújo, Izabela Guimarães Barbosa, Stela Maris Aguiar Lemos, Renan Barros Domingues, Antonio Lucio Teixeira

**Affiliations:** Programa de Pós-Graduação em Neurociências da Universidade Federal de Minas Gerais, Belo Horizonte MG, Brazil.

**Keywords:** migraine, cognition, cognitive impairment

## Abstract

Patients with migraine often report cognitive complaints, especially regarding attention and memory.

**Objective:**

To perform a systematic review of the studies available on cognitive evaluation in patients with migraine.

**Methods:**

We evaluated all articles containing the key words: "Migraine", "Cognition" and "Cognitive Impairment."

**Results:**

The search strategy resulted in 23 articles. Fifteen out of the 23 studies (65.3%) retrieved reported abnormalities on neuropsychological tests in migraine patients, notably tests of memory, attention and information processing speed. Most of the studies showing cognitive changes in migraine were carried out in neurological care facilities. Conversely, among community-based studies, migraine patients were less likely to present cognitive changes.

**Conclusion:**

Patients with migraine, especially those followed at neurology clinics, show an elevated risk of mild changes in several cognitive domains. Further studies with greater methodological refinement are warranted in order to clearly establish whether this cognitive dysfunction is associated with an underlying migraine pathophysiological process.

## Abstract (pt)

Pacientes com migrânea frequentemente se queixam de problemas cognitivos, sobretudo relacionados à memória e à atenção.

**Objetivo:**

Realizar uma revisão sistemática sobre os estudos com avaliação cognitiva em pacientes com migrânea.

**Métodos:**

Foram avaliados todos os artigos que incluíram as palavras-chave "Migraine", "Cognition" and "Cognitive Impairment".

**Resultados:**

Foram encontrados 23 artigos que estudaram desempenho cognitivo em pacientes com migrânea. Dentre estes, quinze (65,3%) relataram alterações em testes neuropsicológicos em pacientes com migrânea, notadamente em testes de memória, atenção e velocidade de processamento. A maioria dos trabalhos que mostraram alterações cognitivas na migrânea foi realizada em serviços neurológicos. Por outro lado, nos estudos de base populacional, essas alterações eram menos comuns.

**Conclusão:**

Pacientes com migrânea, especialmente os acompanhados em serviços neurológicos, apresentam risco aumentado de comprometimento leve em diferentes domínios cognitivos. Estudos com maior refinamento metodológico são necessários para definir claramente se essas alterações cognitivas estão associadas a processo fisiopatológico subjacente à migrânea.

## INTRODUCTION

Migraine is the second most common type of primary headache having a worldwide prevalence of 10-12% in the adult population, being much more prevalent among women.^1,2^ Migraine is also a highly disabling disorder and the leading cause for individuals seeking medical attention at headache and neurological care facilities.^3^

Patients with migraine often report cognitive complaints, particularly concerning deficits of attention and memory. Several studies have addressed cognitive abnormalities in migraine patients outside headache attacks. However, no general consensus has yet been established regarding the cognitive performance of these patients. With the aim of summarizing and reporting the data on cognitive changes in patients with migraine, we carried out a systematic review of the literature.

## METHODS

A systematic review was done on the Medline database. Articles in both Portuguese and English published from 1980 to June 2011 were searched. The key words used were: "Migraine", "Cognition" and "Cognitive Impairment". Original articles involving patients with migraine and neuropsychological evaluation were included in the study. The following data were stratified as follows:

[1] age;
[2] discrimination of studies carried out at neurological care facilities or within the community;
[3] number of patients;
[4] migraine with or without aura;
[5] inclusion of a control group. Studies including patients with other neurologic disorders besides migraine were excluded.

## RESULTS

The search strategy resulted in the retrieval of 23 articles. Seventeen studies (73.9%) evaluated adult patients^4-20^ (Tables 1 and 2) and six (26.1%) evaluated children and teenagers with migraine^21-26^ (Tables 3 and 4). Fifteen studies (65.3%) were carried out at neurological units,^4-14,21-24^ while eight (34.7%) were community-based studies.^15-20,25,26^ Fourteen (60.9%) studies discriminated migraine with and without aura.^5-10,12,13,15-17,19,24,26^ Fifteen out of the 23 studies (65.3%) reported abnormalities on the neuropsychological tests;^4,5,7-11,13,14,17,21,23-26^ ten of these registered memory impairment;^4,5,7-11,17,21,24^ eight detected attention deficit,^5,7-10,17,24,25^ and six reported reduction in information processing speed among patients with migraine.^4,5,9,17,24,26^

Eleven out of 17 studies in the adult migraine population were carried out at neurological care facilities (Table 1).^4-14^ Nine of these studies reported cognitive abnormalities in migraine.^4,5,7-11,13,14^ The most frequent cognitive changes were: memory impairment (7/9),^4,5,7-11^ attention deficit (5/9),^5,7-10^ reduced information processing speed (3/9),^4,5,9^ and executive dysfunction (3/9).^8,11,13^ Among community-based studies (Table 2), only one out of six studies reported cognitive changes.^15-20^ Immediate and late memory impairment, attention deficit, and reduced information processing speed were found in the cited community-based study.^17^

**Table 1:** Studies evaluating adults with migraine in clinical settings.

Article	N of patients/controls	N of patients with aura	Neuropsychological tests	Results
Zeitlin and Oddy, 1984^4^	19/19	N.S.	Stroop Color/Word Test; Trail Making Test; Choice reaction time; Paced Auditory Serial Addition Test; National Hospital Forced Choice Recognition Test for words and faces; Mill Hill Vocabulary Scale	Verbal expression and comprehension, and recognition memory changes and reduction in information processing speed
Hooker and Raskin, 1986^5^	31/15	16	Assessment of own functioning inventory	Recognition memory, verbal expression and comprehension, sustained attention changes and reduction in information processing speed
Bell et al., 1999^6^	20/40	4	Logical Memory (LM I and II); Verbal Association of Word Pairs (VPA I and II); Visual Reproduction (VR I and II); Wechsler Memory Scale – Reviewed; Trail Making Tests A and B; Stroop test; Cubes (WAIS-R); Controlled oral word association test; Paced Auditory Serial Addition Test; Reading Test for Adults	No changes
Le Pira et al., 2000^7^	30/14	14	Boston Visual Exposition Test; Raven's Progressive Matrices; Verbal Fluency – F A S; Rey Complex Figure; Digits (WAIS); Corsi Blocks; California Verbal Learning Test	Verbal and visual memory evocation, sustained attention and visuo-spatial ability changes
Meyer et al., 2000^8^	172/0	39	Mini-Mental State Examination; Cognitive Capacity Screening Examination	Attention, concentration, memory, calculus, capacity to solve problems and judgment changes
Calandre et al., 2002^9^	60/30	10	Wechsler Adult Intelligence Scale; Stroop Test; Black Letters List (Strub); Trail Making Test A and B; Rey Auditory Verbal Learning Test; Wechsler Memory Scale; Rey Complex Figure; Benton Visual Retention Test; Visual Reaction Time; Luria's motor sequence test; Rhythm Test; Poppelreuter's Test; Benton Shape Recognition Test; Benton Facial Recognition Test	Visual-motor processing, reaction time, memory and attention changes
Le Pira et al., 2004^10^	45/0	21	Boston Visual Exposition Test; Raven's Progressive Matrices; Verbal Fluency – F A S; Rey Complex Figure; Digits (WAIS); Corsi Blocks; California Verbal Learning Test	Immediate and late visual memory evocation, learning, verbal supported attention visuo-spatial ability changes
Mongini et al., 2005^11^	23/23	N.E.	Gambling Task; Tower of Hanoi-3; Object Alternating Test	Planning, sequencing skills and working memory changes (preservation)
Pearson et al., 2005^12^	74/74	45	AH4 test; Mill Hill Vocabulary Test; Digit Symbol Substitution Test	No changes
Camarda et al., 2007^13^	45/90	45	Mini-Mental State examination; Token Test; Test d'intelligenza Breve; Trail Making Test Part A and B; Phonemic Fluency; Wisconsin Card Sorting Test	Executive function changes and anxiety increase
Schmitz et al., 20081^4^	24/24	N.E.	Maudsley attention and Response Suppression battery	Reaction time change

N: number; N.E.: not evaluated.

**Table 2:** Studies evaluating adults with migraine in the community.

Article	N of patients/controls	N of patients with aura	Neuropsychological tests	Results
Burker et al., 1989^15^	47/24	20	Halstead-Reitan Neuropsychological Test Battery; Selective Reminding Test; Rey-Osterrieth Complex Figure Test	No changes
Leijdekkers et al.,1990^16^	37/34	11	Groninger Intelligence test; Cubes and codes (WAIS – R); Letter Series Tests; Neurobehavioral Evaluation System (NES)	No changes
Mulder et al.,1999^17^	30/30	10	Neurobehavioral Evaluation System (NES2)	Expression, immediate and late memory, sustained attention changes and reduction in information processing speed
Jelicic et al., 2000^18^	99/1753	N.E.	Letter Digit Substitution Test; Verbal Learning Test	No changes
Gaist et al., 2005^19^	504/857	157	Verbal Fluency – animals; Codes and digits (WAIS); Delayed word recall test	No changes
Mckendrick et al., 2006^20^	29/27	N.E.	Repeatable Battery for the Assessment of Neuropsychological Status	No changes

N: number; N.E.: not evaluated.

Among the six studies on children,^21-26^ four were carried out at neurological units (Table 3) and two within the community (Table 4). Five of these studies reported cognitive changes in children with migraine.^21,23-26^ As observed in adults, the main cognitive changes in children and teenagers were: memory impairment (2/6),^21,24^ attention deficit (2/6),^24,25^ and reduction in information processing speed.^24,26^

**Table 3:** Studies evaluating children and teenagers with migraine in clinical settings.

Article	N of patients/controls	N of patients with aura	Neuropsychological tests	Results
D'Andreaet al., 1989^21^	20/20	N.E.	Raven's Colored Progressive Matrices; Digit Span; Rey Figures; Logical Memory; Ten word learning	Memory change
Haverkamp et al., 2002^22^	37/17	N.E.	Kaufman – Assessment Battery for Children	No changes
Parisi et al., 2010^23^	63/79	N.E.	WISC-R	Total Intelligence Quotient and Verbal Intelligent change
Moutran et al., 2011^24^	30/0	8	WISC-R	Attention, processing speed, memory and perceptual organization changes

N: number; N.E.: not evaluated.

**Table 4:** Studies evaluating children and teenagers with migraine in the community.

Article	N of patients/controls	N of patients with aura	Neuropsychological tests	Results
Waldie et al., 2002^25^	114/739	N.E.	Peabody Picture Vocabulary Test; Illinois Test of Psycholinguistic Abilities – verbal comprehension and expression; Wechsler Intelligence Scale for Children; Word Reading Test	Verbal ability, language comprehension and selective attention change
Riva et al., 2006^26^	48/0	17	Raven progressive Matrices; Digit Span Test; Corsi Span Test; Trail Making Test; Cancellation Test; Computerized Simple Visual reaction time task	Reduction in Information processing speed

N: number; N.E.: not evaluated.

Eight out of 23 articles retrieved reported no cognitive impairment in the tested cognitive domains.^6,12,15,16,18-20,22^

## DISCUSSION

Cognitive function in migraine patients has been reviewed in the present study. Despite mixed results, most studies found that migraine patients have lower cognitive performance than controls. The most frequently reported cognitive changes were impaired visual and verbal memory, reduced information processing speed, executive dysfunction, and attention deficit. The presence of cognitive impairment in patients with migraine reinforces the complexity of this disease, which is not exclusively associated with pain symptoms.

The underlying reason why cognitive changes occur in patients with migraine remains a matter of debate. In a seminal cohort study in New Zealand, 114 patients with migraine and 739 controls were followed for 3 to 26 years.^25^ Migraineurs showed worse performance on verbal tasks and school performance compared to controls without headache or subjects with tension type headache. Interestingly, cognitive functioning was compromised even before the development of headache crises in the future migraine patients.^25^ Frequency of migraine attacks and disease duration did not seem to influence cognitive performance.^7,10,12,16,27^ Taken together, these results suggest that cognitive changes in migraine may be related to the central nervous system dysfunction underlying migraine pathophysiology, and to the result of drugs or pain. It is worth mentioning, however, that cognitive complaints are more evident during the prodrome and headache events.^28,29^

Migraine pathophysiology remains poorly understood. Changes in brain white matter, frequently regarded as incidental, are more common in patients with migraine than controls.^30^ Strong associations between migraine with aura and deep white mater lesions have also been observed.^31^ A study using SPECT reported that patients with migraine showing cerebral hypoperfusion had worse performance on visual and verbal memory tests.^32^ Reduced parietal and frontal gray matter was associated with slowing of reaction time in patients with migraine compared to controls.^33^ Another study showed cognitive abnormalities, particularly in executive functions and attention, in patients with familial hemiplegic migraine with cerebellar atrophy.^34^

Despite the relatively small number of studies and the methodological heterogeneity among investigations, it seems that migraine-with-aura patients display more prominent cognitive changes. Besides exhibiting worse performance on tasks evaluating sustained attention and processing speed,^5,17^ such patients more frequently exhibit anomia and prosopagnosy.^35^ Previous studies have demonstrated that aura seems to be related with cortical functioning interference (cortical spreading depression) and reduced brain perfusion.^36^ Also, unlike migraine without aura, migraine with aura has been associated with increased risk of cerebrovascular disorders.^37^ This could support the argument for migraine with aura being a more deleterious or severe migraine subtype in comparison with migraine without aura, thus explaining worse cognitive performance.

A third of the studies failed to report cognitive changes in migraine. These conflicting reports might result from differences in the analyzed populations and heterogeneity of the neuropsychological tests applied.^38^ Another explanation may be that cognitive dysfunction is seen only in the subset of migraineurs with more severe neurological involvement. This systematic review registered that most community-based studies have not reported cognitive changes in migraineurs. Conversely, the majority of studies conducted in neurological care facilities found cognitive changes. In general, patients referred to tertiary treatment centers show a more serious and/or disabling form of migraine, with high prevalence of comorbid conditions such as depression, possibly explaining the higher frequency of cognitive impairment found in patients evaluated in this setting.

Few studies investigated the presence of cognitive changes in children and teenagers with migraine. In line with the studies involving adult patients, reaction time, visual memory, and verbal memory are impaired in migraine children compared to controls.^21,26^ It is worth mentioning the recent study by Parisi et al. (2010)^23^ which described an inverse correlation between the frequency of attacks and total intelligence quotient scores, verbal intelligence quotient and intelligence performance quotient. Migraine may potentially affect learning and, as a consequence, school performance,^25^ but further studies are warranted to address this issue.

Neurophysiological tools, such as P300, have shown potential usefulness as an indicator of certain features of cognition. P300 seems to correlate with attention, information processing, executive functions such as processing speed, classification of stimuli, ability to establish goals, controlling innate impulses, decision-making, and goal directed organizing and planning.^39-41^ Previous studies with P300 showed that migraine patients had reduced P300 amplitude, longer P300 latency,^42^ and reduced long-term habituation^43^ compared to healthy controls. Future studies with neurophysiological tools such as P300 may contribute to the understanding of the neuropsychological impairment in migraine.

Migraine comorbidities, such as depression and anxiety, can influence cognitive performance. Few studies have evaluated the possible impact of these associated psychiatric disorders in the cognitive functioning of patients with migraine. This is relevant given the high prevalence of these comorbidities in adults and children with migraine.^26,44-51^ In a recent review of neuropsychological functioning in migraine, Suhr and Seng stated it is possible that clinical differences among migraine sufferers, such as medical and psychiatric comorbidities and variables associated with treatment-seeking behavior, may account for the variability in cognitive findings.^52^ Another potential confounding factor is the use of drugs for prophylactic treatment, notably topiramate.^53-55^ While recognizing these points as evident limitations in the studies analyzed, cognitive impairment does not seem to be attributed exclusively to psychiatric comorbidities and drug-related cognitive side effects. Future studies should control for the impact of comorbidities and treatment on cognitive functioning in migraine patients. Furthermore, a better definition of the influence of clinical parameters, such as the severity and frequency of headache attacks, and length of the illness, is warranted.

**Conclusion.** The present systematic review suggests that patients with migraine might present higher risk of cognitive impairment, especially in certain neuropsychological domains such as visual memory, verbal memory, information processing speed, attention, and executive functions. It is uncertain, however, whether this cognitive profile is associated with an underlying migraine pathophysiological process or with the presence of confounding factors such as the use of prophylactic and analgesic drugs or the presence of comorbid conditions such as depression.

As there is still much uncertainty in this field, further studies with greater methodological refinement are warranted in order to establish the clinical definition of cognitive impairment in the evaluation and management of patients with migraine.
